# The End of the Amyloid Era? Evidence for a Paradigm Shift in the Quest to Treat Alzheimer’s Disease

**DOI:** 10.1002/alz70861_108950

**Published:** 2025-12-23

**Authors:** Lloyd Tran, Zung Vu Tran

**Affiliations:** ^1^ Biomed Industries, Inc., San Jose, CA USA; ^2^ MedAware Systems, Inc., Broomfield, CO USA

## Abstract

**Background:**

Over the past two decades, anti‐amyloid beta (Aβ) monoclonal antibodies have dominated Alzheimer’s disease (AD) drug development. While three agents—aducanumab, lecanemab, and donanemab—have received FDA approval, the broader clinical trial record for this drug class raises concerns about efficacy and safety. This study reviews and statistically analyzes trial outcomes across major anti‐Aβ antibodies to determine whether recent approvals mark true clinical progress—or signal the need for a paradigm shift in AD research.

**Method:**

We systematically reviewed published Phase 2 and Phase 3 clinical trials of anti‐Aβ monoclonal antibodies, including aducanumab, bapineuzumab, crenezumab, donanemab, gantenerumab, lecanemab, ponezumab, semorinemab, and solanezumab. We focused on two outcome measures that were most frequently reported ‐ Clinical Dementia Rating Scale–Sum of Boxes (CDR‐SB) and Alzheimer’s Disease Assessment Scale–Cognitive Subscale (ADAS‐Cog). The primary endpoints analyzed were the (CDR‐SB) and (ADAS‐Cog). We use bootstrapping methodology, a machine‐learning statistical procedure that resamples from a single dataset (with replacement) to create multiple samples. A bootstrapping method with 10,000 resamples was used to estimate mean differences and 95% confidence intervals (CI).

**Result:**

All studies were randomized, placebo‐controlled trials with durations of 76 to 105 weeks (median: 78 weeks). Sample sizes ranged from 253 to 1,072 participants per arm. Pooled results showed:

• **CDR‐SB**: mean difference = –0.08 (95% CI: –0.31 to +0.22). The score range for CDR‐SB is 0 to 18

• **ADAS‐Cog**: mean difference = –0.53 (95% CI: –1.03 to +0.22). The score range for ADAS‐Cog is 0 to 70/90.

• In both cases, the confidence intervals included zero, indicating no statistically or clinically significant benefit.

**Conclusion:**

The minimal differences between treatment and placebo—relative to the wide scoring scales—suggest that targeting amyloid plaques may be insufficient as a disease‐modifying approach. These findings underscore the urgent need for alternative strategies. One such approach is NA‐831, Biomed’s novel small‐molecule therapy based on neurogenesis and neuroprotection. Unlike amyloid‐targeted antibodies, NA‐831 aims to restore neuronal function and cognition by stimulating endogenous brain repair mechanisms. These emerging strategies may better align with the complex pathophysiology of AD and represent a promising new frontier in treatment.

